# Macrophage Infiltration Correlates with Genomic Instability in Classic Hodgkin Lymphoma

**DOI:** 10.3390/biomedicines10030579

**Published:** 2022-03-01

**Authors:** Suzana Hančić, Paula Gršković, Slavko Gašparov, Slobodanka Ostojić Kolonić, Mara Dominis, Petra Korać

**Affiliations:** 1 Institute of Clinical Pathology and Cytology, Merkur University Hospital, 10000 Zagreb, Croatia; suzanaparlov@gmail.com (S.H.); gasparovslavko@gmail.com (S.G.); mara.dominis@gmail.com (M.D.); 2 Division of Molecular Biology, Department of Biology, Faculty of Science, University of Zagreb, 10000 Zagreb, Croatia; paula.grskovic@biol.pmf.hr; 3 Department of Pathology, Medical School Zagreb, University of Zagreb, 10000 Zagreb, Croatia; 4 Division of Haematology, Department of Internal Medicine, Merkur University Hospital, 10000 Zagreb, Croatia; ostojic@net.hr; 5 Internal Medicine, Medical School Zagreb, University of Zagreb, 10000 Zagreb, Croatia

**Keywords:** classic Hodgkin lymphoma, genomic instability, CD163, granzyme B, *TP53*

## Abstract

Hodgkin lymphoma (HL) is a biologically diverse group of lymphoid tumors, which accounts for 1% of all de novo neoplasms in the world’s population. It is divided into two main groups: the more common classic Hodgkin lymphoma (cHL) and the less common nodular lymphocyte-predominant Hodgkin lymphoma (NLPHL). cHL is further divided into four subtypes, which differ in morphology and the contents of tumor microenvironment. Macrophages are one of the components of tumor microenvironment known to contribute to creating an immunosuppressive microenvironment, which inhibits the activity of cells expressing granzyme B against tumor cells, even when tumor cells are infected with Epstein–Barr virus (EBV). Our research aimed to explore the association between the specific contents of tumor microenvironment and the genetic anomalies in tumor cells. The presence and the relative percentage of cytotoxic T lymphocytes and macrophages was detected by immunohistochemical staining of the antigens specific for certain cell populations. Fluorescent in situ hybridization was used to detect anomalies in the genome of tumor cells and in situ hybridization was used to detect the presence of EBV. Our results show an association between the number of CD163+ macrophages and the number of *TP53* copies or *BCL6* gene translocation. Patients who had a higher number of CD163+ macrophages infiltrating tumor tissue and three or higher number of copies of *TP53* showed poorer survival. We conclude that the presence of macrophages may contribute to genetic instability in cHL, which drives the progression of cHL and decreases survival of the patients.

## 1. Introduction

Hodgkin lymphoma (HL) is a biologically diverse group of lymphoid tumors of similar morphology and immunophenotype. It accounts for 1% of all de novo neoplasms in the world’s population and is one of the most common occurring diseases among younger adults (age 20–40). Its incidence is also increased in the population over 50 years of age [1]. It is characterized by a low number of tumor cells (only about 1–5% of all cells in tumor tissue) that originate from B lymphocytes, but which have mostly lost their B cell phenotype, including the expression of B cell marker CD20 [2]. Tumor cells are divided into two subtypes: large, multinuclear Reed–Sternberg (RS) cell and mononuclear and multinuclear Hodgkin cells [3]. The origin of RS and Hodgkin cells from B lymphocytes is indicated by the expression of transcriptional factors PAX5/BSAP and MUM1/IRF4, important for development and activation of B lymphocytes [4]; however, both types of cells have been found to express markers characteristic for other leukocyte types [5,6,7]. The expression of various genes in these cells differs from their expression in non-tumor B lymphocytes, but also in various B cell non-Hodgkin lymphomas (B-NHL) and lymphoblastoid cell lines (LCL) [8]. Surprisingly, the deregulated expression of some of these genes remains consistent in different subtypes of HL [8], even though these subtypes display very different phenotypes [2].

According to the classification of the World Health Organization (WHO), HL is divided into two main groups, based on their biological, morphological and clinical characteristics: the more common classic Hodgkin lymphoma (cHL) and the less common nodular lymphocyte-predominant Hodgkin lymphoma (NLPHL), which accounts for only around 5% of all HL cases. The tumor stage (I–IV) is defined by the Ann Arbor (AA) classification, which encompasses both HL and NHL [9], but is additionally described by the German Hodgkin Study Group (GHSG) scale depending on the presence of risk factors (presence of a large mediastinal mass, extranodal disease, high erythrocyte sedimentation rate (ESR) and involvement of three or more lymph node areas) [10]. The Eastern Cooperative Oncology Group (ECOG) scale is used to describe the state of patients depending on their ability to continually care for themselves [11].

Depending on the number of reactive cells infiltrating tumor tissue and the morphology of the tumor cells, cHL is divided into four subtypes. Nodular sclerosis classic Hodgkin lymphoma (NSCHL) is the most common subtype of cHL, accounting for 70% of all cHL diagnoses. It is characterized by collagen bands that surround at least one nodule and Hodgkin and RS cells with lacunar type morphology. It has the best prognosis of all cHL subtypes. The second most common type of cHL is mixed cellularity classic Hodgkin lymphoma (MCCHL), accounting for 20–25% of all cHL cases. It is the most common type of lymphoma in patients infected with human immunodeficiency virus (HIV). The tissue sample is dominated by inflammatory cells (neutrophils, eosinophils, histiocytes and plasma cells) and scattered Hodgkin and/or RS cells, but it lacks the nodular sclerosing fibrosis of NSCHL. If diagnosed early, this type of cHL has almost as good prognosis as NSCHL. Lymphocyte-rich Hodgkin lymphoma accounts for 5% of all HL cases and is morphologically similar to NLPHL, which makes immunohistochemical staining necessary for diagnosing. Somewhat smaller tumor cells are surrounded by small lymphocytes and histiocytes, with mostly absent neutrophils and eosinophils. The prognosis of this type of cHL is similar to the prognosis of NLPHL, though relapses occur more often in patients diagnosed with NLPHL. Lymphocyte-depleted Hodgkin lymphoma is the least common type of cHL, appearing in less than 1% of cases. It is characterized by the large number of RS and Hodgkin cells and a lower number of infiltrating cells. It is most often diagnosed in patients with HIV infection who are also infected with Epstein–Barr virus (EBV). These patients generally have the poorest prognosis of all patients diagnosed with cHL [1].

The contents of the tumor microenvironment (TME) affect not only the morphology of the tumor, but also its clinical behavior. TME in HL consists of different cell types and also various cytokines and chemokines that these cells (and tumor cells) produce (reviewed in [12]). The interactions between tumor cells and the cells of TME create a niche that is suitable for tumor development, which allows tumor cells to avoid immune response [13,14,15]. Two subtypes of helper CD4+ T cells are usually found in largest numbers in close proximity to tumor cells: CD4+ Th2 subtype, which favors the proliferation of B cells, and CD4+CD25+FOXP3+ subtype, known as regulatory T cells (Tregs) [16,17,18]. The main role of Tregs is the maintaining of homeostasis in the immune system by inhibiting potentially pathological immune response [19], but their immunosuppressive effects are often exploited by tumors in order to inhibit the anti-tumor response. Along with tumor cells, Tregs produce IL-10 and TGF-β that have inhibitory effects on CD8+Granzyme B+ cytotoxic T cells and CD57+Granzyme B+ NK cells [20]. The immunosuppressive environment even overcomes the proinflammatory Th1-favoured signaling in EBV+ HL [21,22]. Another type of cells that has a role in promoting tumor development in cHL are tumor-associated macrophages (TAM), recognized by their expression of CD68 (M1 phenotype) and/or CD163 (M2 phenotype), which inhibit anti-tumor response, stimulate angiogenesis and migration of tumor cells. [23] An increased expression of CD68 in HL is associated with a shortened progression-free survival (PFS) and overall survival (OS), as well as with poorer response to treatment and an increased likelihood of relapse after autologous hematopoietic stem-cell transplantation [24,25,26]. The role of non-malignant B cells in cHL is less clear, as is their origin. Their localization might be a consequence of cytokine signaling or it might simply be a remainder of the normal lymph node architecture [15]. It has been observed that some of the morphologically normal cells of TME in HL contain numerical chromosomal aberrations, indicating that the genetic instability occurs not only in tumor cells, but also in the surrounding cells [27].

Genetic instability manifests in different ways in RS and Hodgkin cells: distinct DNA mutations (microsatellite instability), numerical aberrations (chromosomal instability), gains and losses of chromosomal regions and structural aberrations (structural chromosomal instability) [28,29]. DNA mutations are often found in tumor-suppressor genes, rather than in oncogenes [28], but it remains unclear whether these mutations are a consequence of the impaired functions of mismatch repair (MMR) genes [30,31,32]. Some amplifications and deletions common to other malignancies appear frequently, but not always in HL as well as some translocations, but unlike in NHL, no characteristic translocations have been found so far in HL [29]. Numerical chromosomal aberrations have also been found in Hodgkin and RS cells [33,34,35]. Given that some subtypes of cHL are strongly associated with HIV and EBV infections, it is possible that these viruses contribute to the genetic instability of HL.

Infection with EBV has been confirmed in 40% of cHL cases in western countries [36]. Infected tumor cells express EBNA1 (EBV nuclear antigen 1), LMP1 (latent membrane protein 1) and LMP2a (latent membrane protein 2a), which play a part in the pathogenesis of the disease [37,38]. By affecting the anti-tumor response and possibly contributing to genetic instability of tumor cells, EBV infection is an additional factor affecting the pathogenesis of cHL, but its influence on the outcome and the survival of the patients has not yet been exactly defined [26].

The aim of this study was to explore the association between the presence of specific TME components and known chromosomal aberrations representing genome instability in cHL tumor cells, and to assess their effect on patients’ survival.

## 2. Materials and Methods

### 2.1. Patients

A total of 120 samples of cHL from patients consecutively diagnosed in University Hospital Merkur between 2000 and 2009 were analyzed. The tissue samples were reviewed by three experienced hematopathologists (MD, SG and SD) who confirmed diagnoses according to the criteria of the WHO classification [1]. Germinal centers of 8 non-tumor tonsils from patients who were not diagnosed with any hematological disease served as the control group. All tissue samples were fixed in 10% formalin and embedded in paraffin (FFPE).

### 2.2. Tissue Microarray

All FFPE samples were deparaffinized and routinely stained with hematoxylin and eosin (HE). Representative fragments of tissue 5 mm in diameter were extricated and allocated to a previously determined position in a tissue microarray (TMA) block. One block contained fifteen tumor tissue samples and one control sample. The blocks were again embedded in paraffin, cut using a microtome into 2-μm-thick tissue sections and transferred onto adhesive slides.

### 2.3. Immunohistochemical Staining

Tissue microarrays were prepared for immunohistochemical staining. Standard protocols with antibodies most commonly used for selected markers were used. Immunohistochemical staining was carried out using 2-μm-thick tissue sections and was performed after heat induced epitope retrieval (HIER) using polymer-based detection systems EnVision (Dako/Agilent, Santa Clara, CA, USA) according to the manufacturer’s instructions by automated immunostainer (Autostainer Link 48, Dako/Agilent, Santa Clara, CA, USA). Secondary antibodies were conjugated with horseradish peroxidase to enable chromogenic detection upon the addition of 3,3′-diaminobenzidine (DAB). Monoclonal antibodies listed in Table 1 were used as a ready-to-use solution (CD68), at 1:50 dilution (Granzyme B), 1:100 dilution (FOXP3) and 1:40 dilution (CD163). After the staining, the samples were analyzed using Olympus BX51 microscope (Olympus Corporation, Shinjuku City, Tokyo, Japan) at 400× total magnification and Olympus Soft Imaging Solutions (OSIS) software, 2010 version. Immunohistochemical results were evaluated by four independent researchers. In order to evaluate relations of selected cell populations in non-tumor lymphoid tissue, the percentage of cells harboring each specific marker was assessed in the germinal center of tonsils (used as positive controls in immunohistochemical staining). Average percentage of each marker/cell population in the germinal centers was used as a cut-off value, indicating that specific cell populations infiltrating the tumor tissue were more or less present in the tumor tissue than in the corresponding non-tumor tissue of tumor origin.

### 2.4. Fluorescent In Situ Hybridization

Fluorescent in situ hybridization (FISH) was used to detect deletions of 9q34 region, translocations of *BCL2* and *BCL6* and the number of *TP53* copies. In brief, 2-μm-thick tumor tissue samples on adhesive slides (Biognost, Zagreb, Croatia) were incubated in Borg Decloaker ready-to-use solution (Biocare Medical, LLC, Concord, CA, USA) under pressure at 125 °C for two minutes in a Pascal Decloaking Chamber (Dako/Agilent, Santa Clara, CA, USA). Samples were then treated with pepsin (0.1 g/mL, Sigma Aldrich, St. Louis, MO, USA) at 37 °C for 20 min in order to remove proteins. After dehydration through increasing concentrations of ethanol (70%, 85% and 100%) and air-drying for ten minutes, 2 µL of specific probe (that was previously prepared according to the manufacturer’s instructions) was added to samples. The samples were then incubated for 5 min at 85 °C in DakoCytomation hybridizer (Dako/Agilent (Santa Clara, CA, USA)) and left to hybridize over night at 37 °C. Samples were washed the next day in 0.0003% Tween/0.4× SSC solution at 72 °C for two minutes, then in 0.0001% Tween/2× SSC solution at 72 °C for one minute. The samples were covered with 10 µL of Vectashield solution (Vector Laboratories Inc., Burlingame, CA, USA) containing 1.5 µg/mL 4′,6-diamidino-2-phenylindole (DAPI), covered with cover slips and analyzed using Olympus BX51 microscope (Olympus Corporation, Shinjuku City, Tokyo, Japan) at 1000× total magnification and OSIS software, 2010 version by three independent researchers. The results of FISH were defined as either positive or negative. In order for the result to be considered positive, more than 7% of the analyzed 200 morphologically preserved tumor cells had to contain the characteristic changes in detected signals. *BCL2* translocation was analyzed using LSI BCL2 FISH DNA probe, Split Signal (Dako/Agilent (Santa Clara, CA, USA)), while *BCL6* translocation was analyzed using LSI BCL6 Dual Color Break-apart Rearrangement Probe (Vysis Inc., Abbott Molecular, Chicago, IL, USA). *TP53* copy number was analyzed using LSI TP53 SpectrumOrange Probe (Vysis Inc., Abbott Molecular, Chicago, IL, USA).

### 2.5. In Situ Hybridization

In situ hybridization (ISH) was used to detect the presence of EBV in patients’ samples. In brief, 3-μm-thick tumor tissue sections on slides were deparaffinized in xylene three times for two minutes, then rehydrated in decreasing concentrations of ethanol. Samples were then incubated in proteinase K solution (diluted 1:10 with Tris-buffered saline (TBS)) for 20 min, washed in dH2O and dehydrated for one minute in 96% ethanol. After air-drying for ten minutes, EBER (Epstein-Barr encoding RNA) PNA probe (Dako/Agilent (Santa Clara, CA, USA)) was added to tissue samples, which were then covered with cover slips. Slides were then transferred into DakoCytomation hybridizer and incubated at 55 °C for 90 min. After hybridization, slides were washed in washing solution (PNA ISH Detection Kit, Dako/Agilent (Santa Clara, CA, USA)) diluted 1:60 for 25 min at 55 °C and in TBS at room temperature. Anti-FITC/AP (alkaline phosphatase-conjugated antibody to fluorescein) was added to samples and incubated for 30 min in humid conditions at room temperature. After washing in TBS and dH2O, the substrate 5-bromo-4-chloro-3-indolylphosphate (BCIP) and nitroblue tetrazolium (NBT) was added and slides were incubated for 30 min. After washing in dH2O, samples were stained with Nuclear Fast red dye (Dako/Agilent (Santa Clara, CA, USA)). The samples were then dehydrated at 55 °C for five minutes, washed in xylene three times for one minute and covered with mounting medium and cover slips in order to be longer and better preserved. The samples were analyzed using Olympus BX51 microscope (Olympus Corporation, Shinjuku City, Tokyo, Japan) at 400× total magnification and OSIS software, 2010 version by three independent researchers. The results of ISH were defined as either positive or negative.

### 2.6. Statistical Analysis

After the evaluation of stained and hybridized slides, Chi-square test was used to determine the association between the variables. Kaplan-Mayer analysis was used for comparing OS and disease-free survival (DFS) distributions between groups. Statistical analysis was performed with the STATISTICA software, version 13.0 (StatSoft Inc., Tulsa, OK, USA). The level of significance was set at *p* < 0.05.

## 3. Results

### 3.1. Patients’ Clinical Data

General data about the patients are shown in Table 2. Women predominated over men (59.2% vs. 40.8%). The average age of the patients was 34, while the median age was 30. Over 75% of the patients were younger than 40 years of age. In total, 64% of the patients were diagnosed with NSCHL, while the rest were diagnosed with MCCHL. Over 75% of the patients were diagnosed in the early stages of the disease (stages I and II) according to the Ann Arbor (AA) classification, but a share of patients diagnosed with stage II also displayed a number of risk factors, thereby classified as intermediate or even advanced stage on the GHSG scale. Almost all of the patients for whom the data were available scored 1 on ECOG scale, indicating they were fully capable of self-care. Half of the patients displayed B symptoms (fever, drenching night sweats and loss of more than 10% of body weight over 6 months), while bulky disease and bone infiltration were observed in a lesser percentage of cases, 33% and 4%, respectively. Almost all the patients were treated with Adriamycin Bleomycin Vinblastine Dacarbazine (ABVD) as the first line of therapy, following with most patients achieving complete or partial remission. Patients who required a second line of therapy mostly underwent stem cell transplantation and achieved complete remission in most cases. Tumor cells lacked the expression of CD20 in over 90% of cases and expressed CD15 in almost equal percentage of cases. EBV was detected in around 15% of the cases, while the rest of the patients were EBV−. Complete general data were available for 106 patients, while for 14 patients, only sex, age, diagnosis, CD20 and CD15 status and EBV status were available.

The presence of EBV infection was significantly more often observed in patients diagnosed with MCCHL (12/20) compared to patients diagnosed with NSCHL (*p* = 0.014). In total, 40% of all tissue samples were rich in cells expressing granzyme B and were evenly distributed between patients diagnosed with NSCHL (23/48) and MCCHL (25/48). However, only 18 out of 72 samples with a low number of granzyme B+ cells belonged to MCCHL group (*p* = 0.002).

### 3.2. EBV Infection Attracts Cytotoxic T Lymphocytes and Macrophages to TME

**Figure 1:**
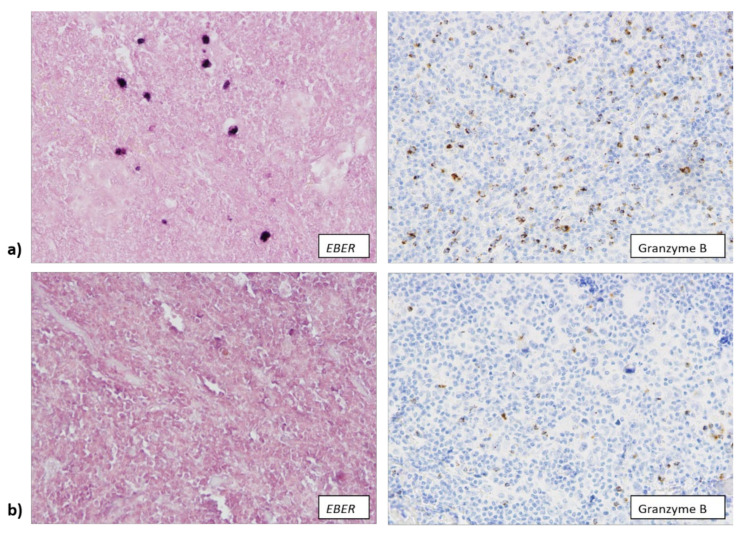
(**a**) Tissue samples in which EBER (Epstein-Barr encoding RNA) mRNA was detected (400× total magnification) contained a larger number of cells expressing granzyme B (400× total magnification). (**b**) The number of granzyme B+ cells (400× total magnification) was significantly lower in samples that were not infected with EBV (400× total magnification).

In accordance with the above-mentioned result, we observed that EBV+ samples more often contained a large number of granzyme B+ cytotoxic T lymphocytes (12/20) (Figure 1a), while EBV− samples often contained a low number of cells expressing granzyme B (64/100) (*p* = 0.046) (Figure 1b). We also observed an association between the presence of EBV infection and the age of the patients, as well as between the number of granzyme B+ cells and the age of the patients. Overall, 8 out of 27 patients (29.6%) older than 40 years of age were infected with EBV, while 81 out of 100 EBV− patients (87.1%) were younger than 40 years of age (*p* = 0.040). Tumor samples from 16 out of 27 patients (59.3%) older than 40 years of age were highly infiltrated with granzyme B+ cells, while a low number of granzyme B+ cells was present in samples of 65.6% (61/93) of patients younger than 40 years of age (*p* = 0.020).

**Figure 2:**
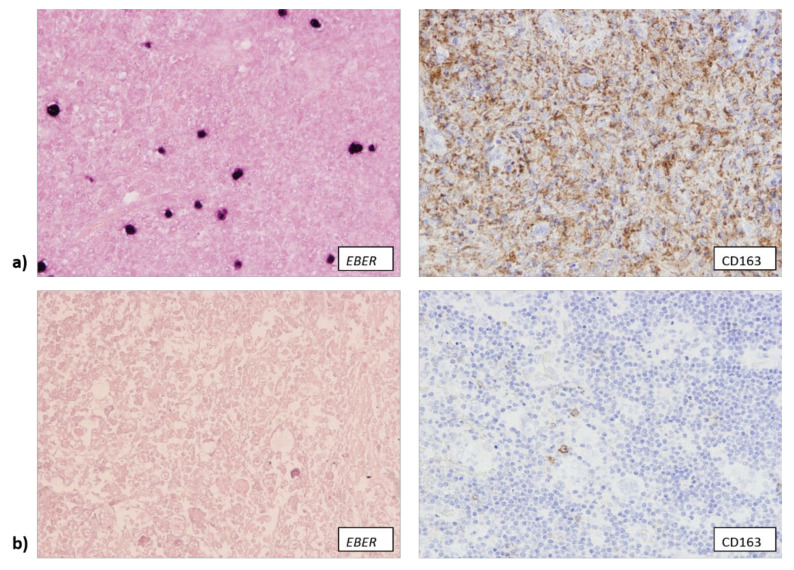
(**a**) Tissue samples in which EBER (Epstein-Barr encoding RNA) mRNA was detected (400× total magnification) contained a larger number of CD163+ cells (400× total magnification). (**b**) EBV-negative samples (400× total magnification) contained a lower number of CD163+ cells (400× total magnification).

Similarly, EBV+ samples were more often rich in CD163+ macrophages (14/18) (Figure 2a), while 59 out 96 EBV− samples contained a low number of CD163+ cells (*p* = 0.002) (Figure 2b).

**Figure 3:**
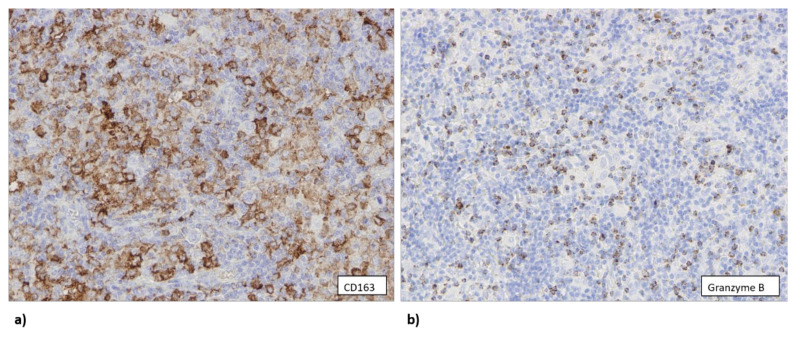
(**a**) Sample with a high number of CD163+ cells (400× total magnification). (**b**) The same sample as on panel (**a**) that also contained a high number of expressing granzyme B (400× total magnification).

We also observed that EBV− samples generally contained a lower number of CD68+ macrophages (79/100), but less than half of EBV+ samples contained a large number of CD68+ macrophages (9/20) (*p* = 0.024). Samples that contained a high number of CD68+ cells also contained a high number of CD163+ cells (*p* = 0.002), but this association between the number of CD68+ cells and CD163+ cells was slightly less straightforward when samples were split into three groups: low number (less than 5% of the overall number of cells within the tumor tissue), intermediate number (5–25% of the overall number of cells within the tumor tissue) and high number (over 25% of the overall number of cells within the tumor tissue). While most of the samples with a high number of CD68+ cells also contained a high number of CD163+ cells (27/29), in 29 out of 40 samples with less than 5% of CD68+ cells an intermediate number of CD163+ cells was detected (*p* < 0.001). Samples that were rich in granzyme B expressing cells also mostly contained intermediate or high numbers of CD68+ and CD163+ cells (*p* < 0.001), showing a strong association between the presence of cytotoxic T lymphocytes and macrophages, possibly conditioned by the EBV infection (Figure 3). We did not observe any association between EBV infection and the presence of FOXP3+ cells, nor between the presence of FOXP3+ cells and other components of TME.

### 3.3. The Number of CD163+ Cells Is Associated with Higher Copy Number of TP53 Gene

**Figure 4:**
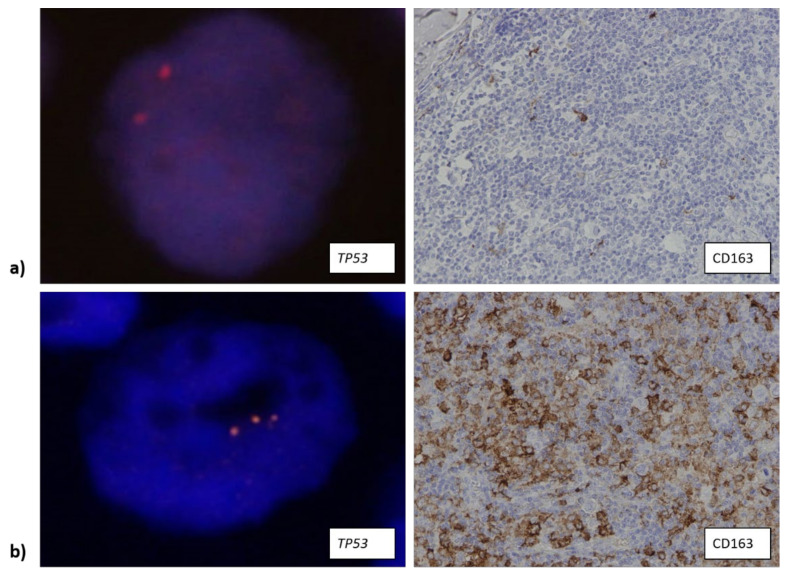
(**a**) Tumor samples that contained two or less copies of the *TP53* gene (1000× total magnification) were infiltrated with a lower number of CD163+ macrophages (400× total magnification). (**b**) Three or more copies of the *TP53* gene (1000× total magnification) were associated with a higher number of CD163+ cells infiltrating the tumor tissue (400× total magnification).

Data about the number of copies of *TP53* gene were available for 84 samples, out of which 30 samples contained three or more copies of *TP53*. Around 75% of samples that had a low number of CD163+ cells contained only one or two copies of *TP53* (Figure 4a). Additional copies of *TP53* were associated with a larger number of CD163+ macrophages in a tumor sample (Figure 4b) (*p* = 0.028).

**Figure 5:**
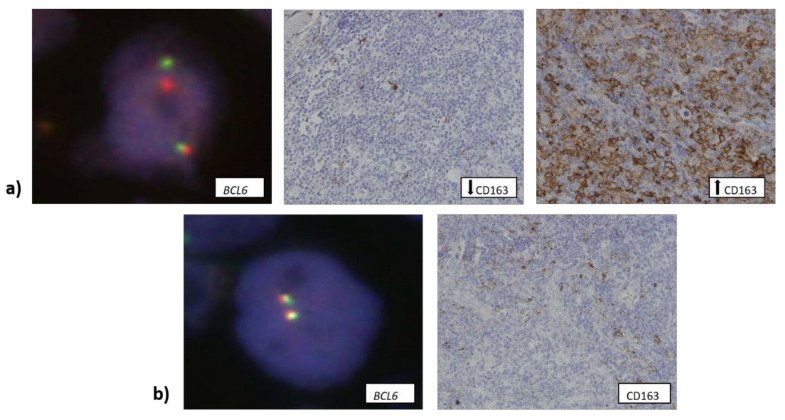
(**a**) Samples that contained a low or a high number of CD163+ cells (400× total magnification) showed *BCL6* aberration (shown as split-apart signal, 1000× total magnification). (**b**) Samples that contained an intermediate number of CD163+ macrophages (400× total magnification) showed *BCL6* translocation significantly less often (1000× total magnification). Down arrow: low number of CD163+ macrophages (less than 5% of the overall number of cells within the tumor tissue); up arrow: high number of CD163+ macrophages (over 25% of the overall number of cells within the tumor tissue).

Data about *BCL6* translocation were available for 114 samples. Only 11 samples (~10%) contained this translocation, but it was more often observed in samples with either a low or high number of CD163+ cells (Figure 5a) (27.3% and 54.5%, respectively) compared to samples that contained an intermediate number of CD163+ cells (Figure 5b) (*p* = 0.016).

### 3.4. CD163+ Macrophages and Additional TP53 Copies Affect the Patients’ Survival

**Figure 6:**
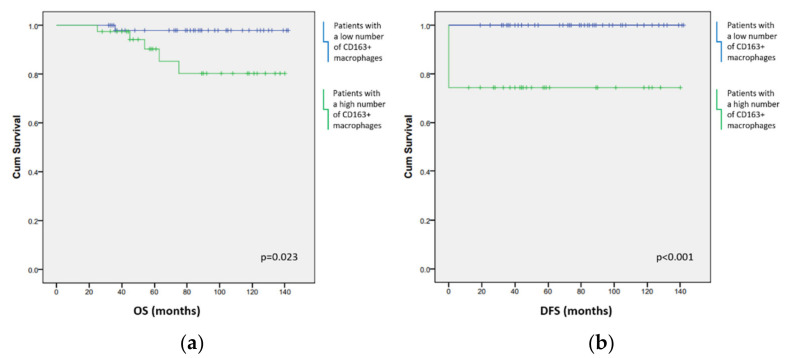
(**a**) A higher number of CD163+ cells contributed to poorer overall survival (OS) of the patients. (**b**) Patients who had a higher number of CD163+ cells also had worse disease-free survival (DFS). Blue line: patients with a lower number of CD163+ macrophages infiltrating tumor tissue; green line: patients with a higher number of CD163+ macrophages infiltrating tumor tissue.

For the evaluation of patients’ survival, we assessed overall and disease-free periods in relation to specific components of the microenvironment and specific genome change. A large number of CD163+ macrophages contributed to poorer OS (*p* = 0.023) (Figure 6a) and DFS (*p* < 0.001) (Figure 6b).

**Figure 7:**
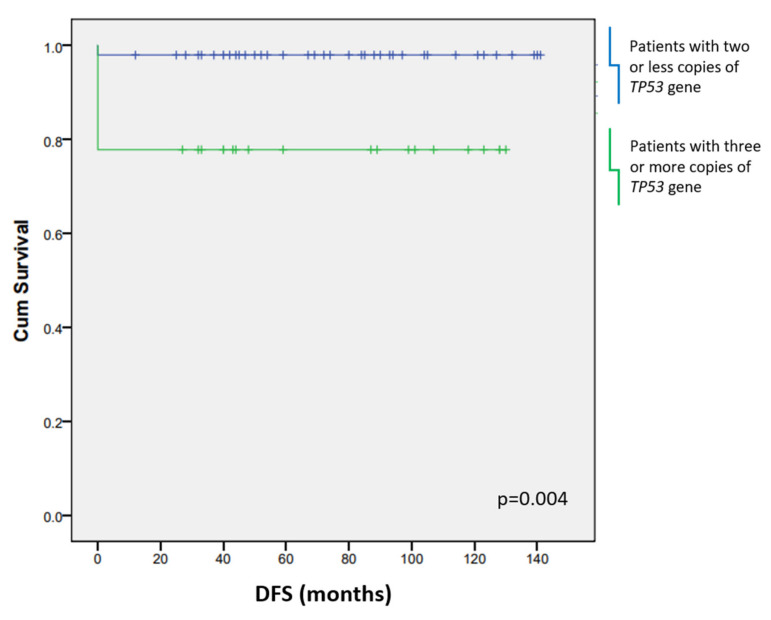
Poorer disease-free survival (DFS) was observed in patients who had three or more copies of the *TP53* gene. Blue line: patients with two or less copies of the *TP53* gene; green line: patients with three or more copies of the *TP53* gene.

DFS of the patients was also affected by the presence of three or more copies of *TP53* (*p* = 0.004) (Figure 7). EBV infection did not affect the length of the patients’ survival nor the outcome of the disease.

## 4. Discussion

The majority of the patients in our cohort were younger than 40 years of age, in accordance with the observation that cHL usually appears in the younger population [1]. The statistically significant association between the age of the patients and their EBV status, which we observed, can be explained by the loss of immunological control over latent infection in older patients [39,40]. A high number of granzyme B+ cells appeared more often in samples from older patients, but unlike in the research by Oudejans et al. [41], neither granzyme B+ cells nor age of the patients had prognostic value. Our results also match previous findings that EBV infection is more common in patients diagnosed with MC subtype of cHL compared to patients diagnosed with NS subtype [42]. Given that infection with EBV stimulates an immune response that consists largely of CD8+ cytotoxic T cells [43], it is expected that the infection with EBV contributes to the observed difference in the expression of granzyme B between the two subtypes. Higher numbers of cytotoxic T lymphocytes and macrophages are found in higher AA stages of B-NHLs [44], but we did not observe dependence of the number of these cells on AA stage in cHL. Our results support the findings by Jakovic et al. [45], that the number of both lymphocytes and monocytes/macrophages affect the behavior of cHL, but while we observed an association between the numbers of these cell types, only the number of CD163+ macrophages affected the outcome of the disease in our cohort.

Genetic abnormalities of *TP53*, including gene amplifications/deletions, are associated with poorer survival of patients diagnosed with B-NHL [46]. They are less common in HL and not associated with the presence of EBV [47]. However, we found that samples that contained *TP53* amplifications also contained a high number of CD163+ macrophages. Given that macrophages can create a mutagenic environment through the release of free radicals [48], their presence could be a contributing factor to genetic instability of RS and Hodgkin cells. We also observed that the number of CD163+ macrophages (but not CD68+ macrophages) is associated with the presence of *BCL6* translocation, with *BCL6* translocation least often observed in the samples with an intermediate number of CD163+ macrophages. This finding can be linked to the results obtained by Werner et al., who observed better survival of cHL patients with an intermediate number of tumor-infiltrating CD163+ macrophages compared to patients who had a high or low number of CD163+ macrophages infiltrating the tumor tissue [26]. Tamma et al. observed that the frequency of *BCL6* translocation negatively correlated with p53 positivity in breast cell carcinoma [49], but it would be worth exploring whether p53+ RS and Hodgkin cells that contain *BCL6* translocation would behave in the same manner, especially in regard to *TP53* amplifications and the presence of macrophages we found in cHL.

The presence of macrophages has been associated with shortened survival in patients with cHL [25,26,50,51,52,53], but the results differ based on the specific marker that was being detected. Similar to Tudor et al. [52], we observed the effect of CD163+ macrophages on the survival of the patients, but there was no statistically significant difference in the survival of the patients dependent on the number of CD68+ macrophages. An explanation for these results might be that CD68 is expressed on a much wider spectrum of macrophages, while CD163 is more specific for TAM polarized towards M2 phenotype [23,54]. Aside from CD163+ macrophages, higher number of *TP53* copies was also associated with poorer survival. Newman et al. found that the lack of *TP53* abnormalities is associated with the better survival as well, but their results were obtained from pediatric cases of B-NHL and also explored *TP53* mutations and deletions [46], rather than *TP53* amplifications. Since both HL and B-NHL originate from B cells, the effect of *TP53* abnormalities on tumor progression could further extend our understanding of the behavior of lymphoma originating from B lymphocytes.

## 5. Conclusions

Our results show that macrophage infiltration may contribute to genetic instability of tumor cells in cHL, thereby driving the progression of cHL and decreasing the survival of patients.

## Figures and Tables

**Table 1:** Analyzed cell markers, antibodies used for their detection on cells of a specific type and cut-off values for each marker.

Marker	Antibody Clone/Manufacturer	Type of Cells	Cut-Off Value
Granzyme B	GrB-7/Dako/Agilent (Santa Clara, CA, USA)	Cytotoxic T cells and natural killer cells	30 cell per high power field
FOXP3	236A/E7/Abcam (Cambridge, UK)	Regulatory T cells	30% of the overall number of cells within the tumor tissue
CD68	PG-M1/Dako/Agilent (Santa Clara, CA, USA)	M1 macrophages	25% of the overall number of cells within the tumor tissue §
CD163	MRQ-26/Cell Marque (Rocklin, CA, USA)	M2 macrophages	25% of the overall number of cells within the tumor tissue §

§ Group with the number of detected cells lower than the cut-off value was additionally split into two: low number (≤5% of the overall number of cells within the tumor tissue) and intermediate number (over 5% and less than 25% of the overall number of cells within the tumor tissue).

**Table 2:** Clinical data of the patients.

Characteristic	Category	*N*	%
Sex	Male	49	40.8
	Female	71	59.2
Age	≤40	93	77.5
	>40	27	22.5
Histology	NSCHL	77	64.2
	MCCHL	43	35.8
Ann Arbor	I	12	11.3
	II	69	65.1
	III	17	16
	IV	8	7.6
GHSG	Early stage	24	22.7
	Intermediate stage	49	46.2
	Advanced stage	33	31.1
ECOG	1	103	97.2
	2	2	1.9
	3	0	0
	4	1	0.9
B symptoms	+	52	49.1
	−	54	50.9
Bulky disease (>7.5 cm)	+	35	33
	−	71	67
Bone marrow infiltration	+	4	3.8
	−	102	96.2
First therapy	ABVD	103	97.2
	Other	3	2.8
Response to first therapy	Complete remission	95	89.6
	Partial remission	9	8.5
	Disease progression	2	1.9
Relapse	+	12	11.3
	−	94	88.7
Second therapy	Stem cell transplantation	21	19.8
	Other	2	1.9
	None	83	78.3
Response to Second therapy	Complete remission	16	69.6
	Partial remission	5	21.8
	No response	1	4.3
	Unknown	1	4.3
CD20	+	9	7.5
	−	111	92.5
CD15	+	109	90.8
	−	11	9.2
EBV-ISH	+	20	16.7
	−	100	83.3

NSCHL: nodular sclerosis classic Hodgkin lymphoma; MCCHL: mixed cellularity classic Hodgkin lymphoma; GHSG: German Hodgkin Study Group; ECOG: Eastern Cooperative Oncology Group; ABVD: Adriamycin Bleomycin Vinblastine Dacarbazine; EBV-ISH: Epstein-Barr virus in situ hybridization.

## Data Availability

Not applicable.
